# Feasibility of 12-lead ECG reconstruction at rest and during adenosine stress perfusion inside a 3-Tesla MR scanner

**DOI:** 10.1007/s10554-026-03772-z

**Published:** 2026-07-21

**Authors:** Debbie Falconer, Maroua Mehri, Mohammed Bhiri, Matthew Webber, Isabelle Whitmore, Emma Martin, Lorena Topala, Giovanni Vagliani, Franca Morselli, Haytham Shah, Swapnanil De, Thomas A Treibel, Michele Orini, Nish Chaturvedi, Iain Pierce, Peter Kellman, Pier D Lambiase, James C Moon, Alun D Hughes, Freddy Odille, Guillaume Calmon, Gabriella Captur

**Affiliations:** 1 UCL Institute of Cardiovascular Science, University College London, London, UK; 2 Epsidy, Rue du Morvan, Vandoeuvre-les-Nancy, France; 3 Barts Heart Centre, West Smithfield, London, UK; 4 UCL Medical School, University College London, London, UK; 5 Chenies Mews Imaging Centre, The Roger Williams Building, London, UK; 6 King’s College London, Strand, London, UK; 7 IADI Laboratory, Institut National de la Santé et de la Recherche Médicale, Rue du Morvan, Vandoeuvre-les-Nancy, France; 8 National Heart, Lung, and Blood Institute, National Institutes of Health, Bethesda, MD USA; 9 The Inherited Cardiac Conditions Unit, Royal Free London NHS Foundation Trust, London, UK; 10 Institute of Cardiovascular Science, University College London, Gower Street, London, WC1E 6BT UK

**Keywords:** Adenosine, Cardiovascular magnetic resonance, ECG, In-bore electrocardiogram, Cardiovascular monitoring, Stress ECG

## Abstract

*Background* Electrocardiographic (ECG) devices in magnetic resonance (MR) scanners have a narrow bandwidth and suffer from signal distortion, preventing measurement of QRS/ QT durations, or detection of ST/ T-wave changes during ischaemia. We integrated an external device with novel post-processing software into a 3 Tesla (T) scanner to assess feasibility and accuracy for reconstructing 12-lead ECGs prior to and during adenosine infusion. *Methods* Reference 12-lead ECGs were first recorded outside the MR-environment. Using three MR-safe electrode patches, in-bore ECGs were then recorded prior to and during adenosine infusion. Post-processing removed artefacts and 12-lead ECGs were reconstructed from the raw signal using a subject-specific matrix. In-bore and reference ECGs were compared using Pearson’s correlation coefficient on the PQRST waveform and by manual measurements of QRS and QT durations. Adenosine-induced ischaemic changes were compared with corresponding territories on stress perfusion maps. *Results* In 26 participants (70.8 ± 13.0 years; 34.6% female), in-bore ECGs were recorded without additional scan time. Reconstruction took < 2 min per participant. In-bore and reference ECGs were strongly correlated (*r* = 0.81, interquartile range 0.72–0.88), and mean differences in QRS and QT durations were small (-12.5 ms [95% limits of agreement − 42.5, 17.5 ms], and − 5.5 ms [-54.6, 43.6 ms] respectively). Ischaemic ECG changes during adenosine predicted stress myocardial blood flow (MBF) < 1.75 ml/g/min with sensitivity 91.7% (95% confidence interval 66.7, 100.0), specificity 59.3% (39.6, 77.6), positive predictive value (PPV) 33.3% (11.4, 57.7), negative predictive value (NPV) 97.0% (90.0, 100.0) and area under curve 0.74 (0.61, 0.86). *Conclusion* In this proof-of-concept study, in-bore ECGs correlated closely with reference ECGs. In-bore ECGs during stress perfusion have high specificity and NPV, but low sensitivity and PPV for predicting stress MBF < 1.5 ml/g/min. This may hold value for improving R-peak detection, patient monitoring and stress perfusion map interpretation.

**Supplementary Information:**

The online version contains supplementary material available at https://doi.org/10.1007/s10554-026-03772-z.

## Introduction

12-lead electrocardiograms (ECGs) recorded in the magnetic resonance (MR) bore could offer a crucial link between cardiac electrophysiology and mechanical function. However, due to constraints posed by the MR-environment, current built-in MR-ECG systems are insufficient for measuring parameters such as QRS and QT duration or detecting ischaemic changes in ST segments or T waves. Despite being essential for gating cardiovascular MR (CMR) scans (1), and for monitoring certain groups (such as intubated patients (2) or those with cardiac implantable electronic devices (3)) even basic R peak detection is frequently unreliable, leading to suboptimal patient monitoring, ECG mis-triggering and degraded image quality. High-quality ECGs could improve patient safety and image acquisition, support MR-guided interventions and offer new insights into emerging fields such as electrocardiographic imaging (4) and neurocardiology. However, several physical and technical obstacles must first be overcome.

Firstly, in-bore ECG signal is affected by the magnetohydrodynamic (MHD) effect (5) – a voltage induced by separation of charged ions in the blood due to the Hall effect, greater at higher field strengths (6). This effect is maximal during ventricular systole, resulting in peak artefact in the ST segments. MHD also worsens with fluctuations in heart rate and cardiac output (7), further complicating interpretation of ECGs acquired during ischaemia testing.

ECG devices currently built into MR-scanners acquire data with a narrow bandwidth (0.5**–**20 Hz) and the resultant ECG trace is considered to be of monitoring quality, allowing heart rate analysis only. Features such as ST segment or T wave changes during ischaemia require diagnostic ECGs with an acquisition bandwidth of 0.05**–**150 Hz (8). Occasionally, gross ST segment deviation may still be visible on a narrower-bandwidth 3-lead trace, but such changes cannot be localised to conventional anatomical territories, which still requires 12-lead ECGs. Filtering can improve signal quality at the expense of altering signal waveform. For example, higher low-frequency filters can improve QRS detection by suppressing lower frequency MHD and respiratory motion (9), but increase distortion of ST segments and T waves (8).

Furthermore, certain hardware is deemed unsafe. Radiofrequency (RF) fields can result in heating of long cables and potential burns, so short ECG cables are generally used (10). Current MR-systems also use fewer electrodes with non-conventional lead placement, meaning that even if arrhythmic or ischaemic changes could be detected, they cannot be localised to traditional clinical territories. Additionally, current systems often use high impedance leads to attenuate induced currents, which can reduce signal amplitude and increase noise (11).

Previous work showed that ST segments could not be accurately correlated with out-of-bore ECGs above 70 millitesla (mT) (12). Given that standard clinical 1.5 T and 3 T MR scanners operate at field strengths 21–43 times greater than this, for in-bore ECG recording to be viable, major alterations to hardware and/or signal post-processing will be required.

We integrated an external ECG device with novel post-processing software into a clinical 3 T scanner. The aims of this proof-of-concept study were to:

i) determine the feasibility of in-bore 12-lead ECG recordings with our system prior to and during adenosine infusion at 3 T;
ii) compare ECG morphology and measurements between in-bore and out-of-bore reference 12-lead ECGs;
iii) determine the reproducibility of in-bore ECG measurements;
iv) determine the performance of in-bore ECG ST/ T wave changes for detecting inducible ischaemia during adenosine stress perfusion, using qualitative and quantitative stress perfusion mapping as the reference standard.

## Methods

### Population and study design

**Fig. 1:**
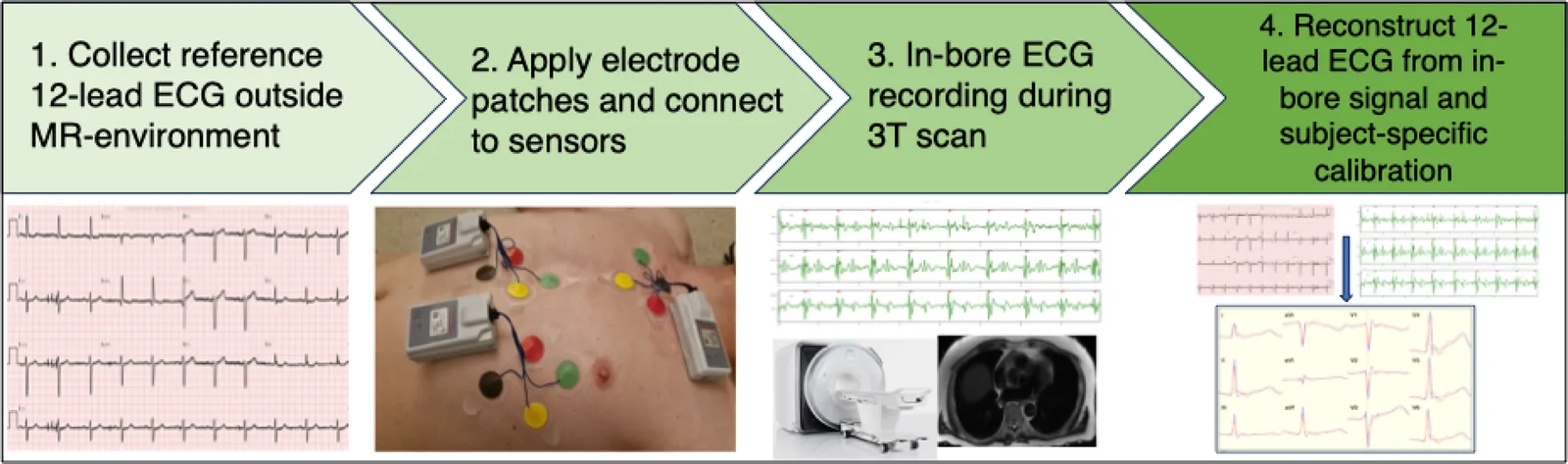
Workflow for in-bore ECG recording and reconstruction. Participants first undergo 12-lead ECG recordings outside the scanner bore with conventional electrode placement. Subsequently, electrode patches, sensors and leads are connected before the participant undergoes CMR at 3 Tesla. In-bore recordings and reference ECGs are used to reconstruct in-bore 12-lead ECGs. *ECG*,* electrocardiogram; CMR*,* cardiovascular magnetic resonance*

26 participants were prospectively recruited as follows: 20 older age participants from a population-based cohort; 2 younger healthy volunteers; 4 patients with known inducible perfusion defects. The older age volunteers were recruited from MyoFit46–the cardiovascular sub-study of the Medical Research Council (MRC) National Survey of Health and Development (NSHD, Research Ethics Committee [REC]: 19/LO/1774, National Clinical Trial [NCT]: NCT05455125) (13). The younger healthy volunteers were recruited through advertising channels to staff at University College London (UCL) and patients with perfusion defects were recruited following referral from their treating physician as part of an ongoing research study (REC 328944, NCT06035107). Exclusion criteria that precluded safe conduct of stress CMR were claustrophobia, inability to provide informed consent, pregnancy, breast-feeding, presence of cardiac implantable electronic devices (excluding loop recorders), high degree atrio-ventricular block, severe or unstable asthma, chronic kidney disease stage 4 or 5 and other standard absolute contraindications to MR scanning. The study workflow is described in Fig. 1.

Sample size calculations were conducted using Fisher’s z-transformation approach for intraclass correlation coefficients (ICC) hypothesis testing (14). A minimum of 21 participants was required to detect an ICC of 0.90, with a lower limit of precision of 0.80 (one-sided significance level of 0.05) for comparing in-bore with out-of-bore ECG metrics. A 20% dropout margin was included to account for potential data loss arising from patient motion or software pipeline failure. Therefore, a total of 26 participants were planned for recruitment to ensure adequate power.

### Reference 12-lead ECG recording (outside the MR-environment)

All participants first underwent reference standard 12-lead ECG recording (10 electrodes in conventional placement) outside the MR-environment at rest (i.e., with no adenosine) in the supine position using a PC-based ECG monitor (Cardioperfect Workstation, Welch Allyn, New York, USA). Digitised ECGs were stored as Standard Communications Protocol-ECG and portable document format files.

To allow direct comparison of in-bore and out-of-bore ECGs during ischaemia testing (and to determine if changes in ST segments occurred as a result of the MR-environment rather than true inducible ischaemia), 12-lead ECGs were recorded in the younger healthy volunteers during an adenosine infusion (140 mcg/kg/min for 4 min) outside the MR-environment following the same protocol used during the CMR scan. Recordings were taken at the start (0–10 s), midway through (180–190 s) and the end of the infusion (230–240 s).

### Hardware for in-bore ECG recording

Three fibre-optic cables were installed in the MR-room prior to study commencement. The cables, bound together and wrapped in a sleeve for ease of use, passed through a penetration hole into the adjacent MRI control room where they connected to an electronics signal module and laptop equipped with bespoke recording software provided by collaborators Epsidy (Vandoeuvre-les-Nancy, France).

**Fig. 2:**
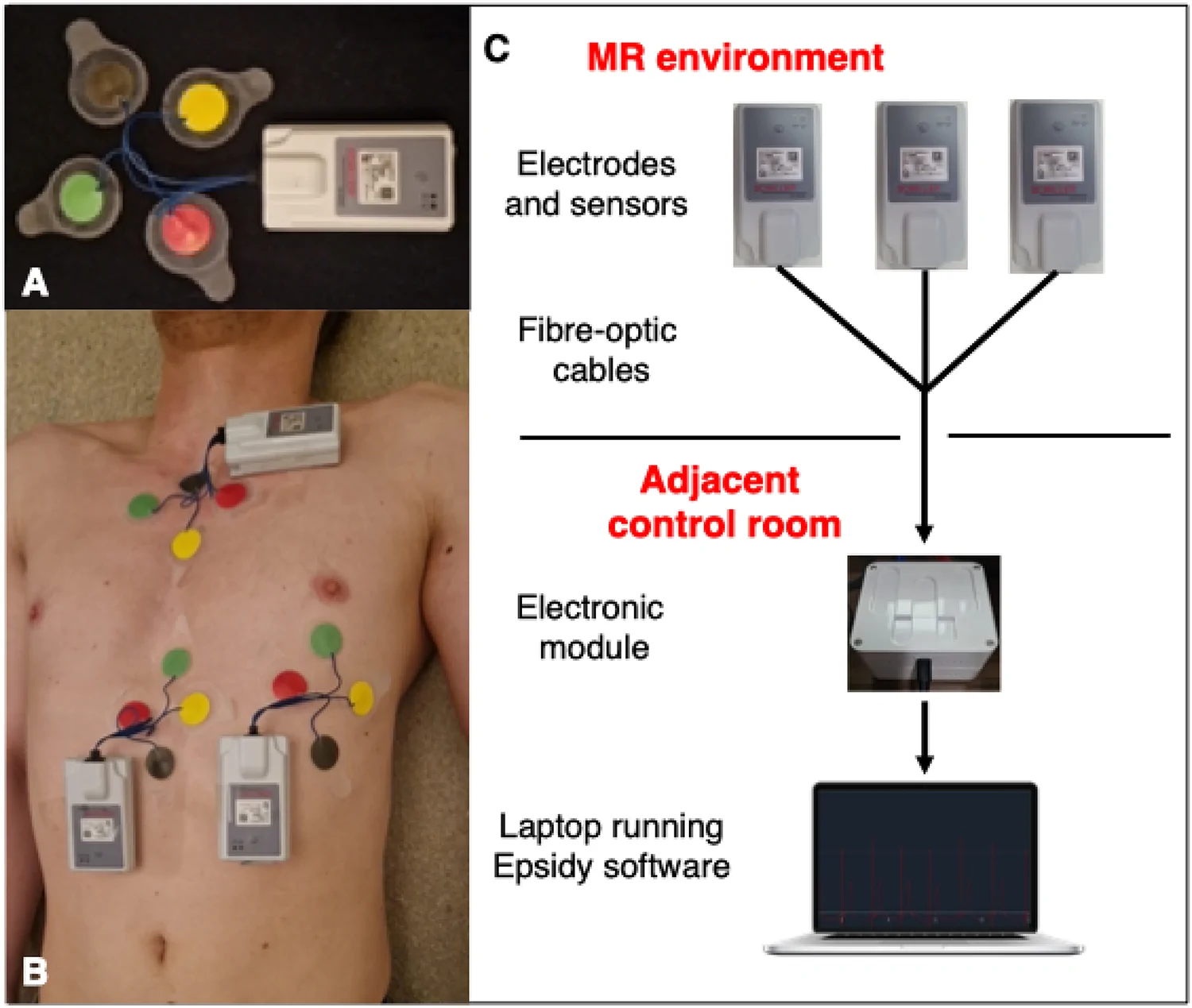
Hardware required for in-bore ECG recording. **A**) Example CMR-safe electrode patch with 3 measurement electrodes and 1 reference (black) electrode. **B**) Electrode configuration on a participant’s chest (if female, lateral electrode positioned under the breast). Each electrode connects to a sensor (grey box) via a short lead for signal amplification and digitalisation. Short leads prevent overheating, minimising the risk of burns. **C**) Hardware set-up inside and out of the MR environment. Cables are passed through a penetration hole to the adjacent control room

Prior to each scan, three MR-safe ECG sensors (Schiller Medical, Wissembourg, France, Fig. 2A), each consisting of 1 reference and 3 measurement electrodes, were applied to the chest of participants (configuration Fig. 2B). Each sensor provided three bipolar ECG leads. In patients with excess chest hair, shaving was performed to optimise electrode contact. Each sensor was connected via a short lead to a front-end ECG module for signal pre-amplification and digitalisation, which provided raw ECG signals at a sampling frequency of 16 kHz (15) (ADAS1000BCPZ, [Analog Devices Inc, Norwood, MA, USA] configured for an acquisition bandwidth of 0 Hz − 3.5 kHz, 18-bit resolution, 6.54 uV/LSB, usable input dynamics of ± 670 mV). Once the participant was positioned on the MR-table, each module was connected to one of the pre-installed fibre-optic cables and secured with medical tape (Fig. 2C) after which the anterior chest coil was placed on top, and the MR-bed and patient moved to the isocentre.

Electrodes were removed and disposed of following the scan. The cables were wound together and stored safely at room temperature between uses while the electronic modules were stored in the MRI control room for recharging.

### CMR scan

All participants were scanned using a 3 T (slew rate 200 T/m/s, gradient strength 80 mT/m) MRI system (Magnetom Prisma, Siemens Healthineers, Erlangen, Germany) operating VE11C-SP01, with an 18-channel phased-array chest coil and spine array (up to 24-elements) equipped with an external reconstruction server running Gadgetron (Linux box, 16–24 processors) (16).

MyoFit46 participants underwent a comprehensive CMR protocol that included quantitative stress perfusion imaging with adenosine (13). Healthy volunteers and patients with known perfusion defects underwent the same quantitative stress perfusion imaging with adenosine, but an otherwise abridged scan protocol (which included localisers; 2-, 3- and 4-chamber and LV [left ventricular] short-axis [SAX] cines). Cines were acquired using conventional breath-held retrospectively ECG-gated balanced steady state free precession (bSSFP) sequences (flip angle 50°, temporal resolution 30 ms, default field of view 380 mm x 285 mm adjusted to body size). All participants were fitted with two cannulas (one in each antecubital fossa) prior to CMR scanning to permit separate infusions of adenosine and GBCA (gadoterate meglumine [Dotarem, Guerbet, France]) and reduce the risk of bolus overlap between GBCA and adenosine. Adenosine was infused at 140 mcg/kg/min for 4 min. Immediately after stopping the adenosine infusion, stress perfusion images were acquired using a free-breathing motion-corrected (MOCO) saturation-recovery bSSFP dual sequence (17), covering 3 LV SAX slices across each heartbeat.

Peripheral brachial and central aortic blood pressure (BP) measurements were performed, on the right arm, directly in the scanner bore, at baseline, peak adenosine stress and recovery using an oscillometric blood pressure machine (Cardioscope II BP+, USCOM, Sydney, Australia). Central BP measurements were derived from analysis of supra-systolic pressure waveforms calibrated to brachial artery BP (18). ECG-triggered gating was performed by the scanner’s built-in 3-lead ECG system, independently of our 12-lead reconstruction pipeline, which was applied retrospectively and offline.

### In-bore ECG recording

ECGs were recorded prior to starting adenosine for 30 s (termed the pre-adenosine or ‘rest’ in-bore ECG), and during the adenosine infusion as follows: at the start (0–10 s), midway through (180–190 s) and at the end of the infusion (230–240 s). Additional time taken to set up the recording equipment was measured for each participant to quantify the impact on clinical CMR patient workflows.

A detailed explanation of the signal post-processing and 12-lead ECG reconstruction can be found in the Supplementary appendix.

### Data analysis and statistical methods

Statistical analyses were performed using R (version 4.1.2). Data were visually assessed for normality using histograms and Q-Q plots. Continuous variables were expressed as mean  ± 1 standard deviation (SD) (normal distribution) or median and interquartile range (IQR) (non-normal distribution), and categorical variables as counts and percentages.

Pearson’s correlation coefficient (*r*) measuring the correlation of the PQRST waveform between in-bore and reference ECGs was calculated for every lead in all participants. *r* measured the correlation between the in-bore signal (a median signal from ten cardiac cycles) and the reference out-of-bore signal.

A non-parametric bootstrap approach (10000 resamples) was applied to compare mean *r* values between unpaired groups (males vs. females, body mass index [BMI] < 30 vs. > 30 kg/m^2^). The 95% confidence interval (CI) for the mean difference (MD) was estimated using the percentile method. Statistical significance was based on whether the CI included zero.

For each participant, QRS and QT intervals were measured manually on the lead with the highest correlation. MDs were calculated (19) and agreement assessed using Bland-Altman plots. Two-way mixed, single measures intraclass correlation coefficient (ICC) (3, 1) was used to assess reliability within each subject. Statistical significance was defined as a *p* value < 0.05 and presented alongside either SD or CI as appropriate. Descriptors of strength of association for *r* and ICC were: <0.5 = weak; 0.5–0.75 = moderate; >0.75 = strong.

PR intervals were not measured, as P waves were not reliably discernible consistent with previous experiments at 3 T (20).

ECGs recorded during adenosine were serially analysed for evolving ischaemic changes, defined as new T wave inversion/flattening or ≥ 1 mm ST depression or elevation in ≥ 1 lead per anatomic territory. Leads were classified into anatomical groups as follows: lateral = I, aVL, V5, and V6; inferior = II, III, and aVF; anteroseptal = V1, V2, V3 and V4 (21).

Stress myocardial blood flow (sMBF) from quantitative perfusion maps was analysed as per the American Heart Association (AHA) model (22) (excluding segment 17). Segments were defined by the following anatomic territories: anteroseptal: 1,2,7,8,13,14; inferior: 3,4,9,10,15; lateral: 5,6,11,12,16. Absolute and rate pressure product normalised (RPP_N_) sMBF were calculated inline from the perfusion maps, where each pixel encoded sMBF in ml/g/min. sMBF was calculated per segment and averaged across 16 AHA segments to represent mean global LV values. Each scan was appraised by a Level II (D.F.) and Level III accredited reader (G.C.), both blinded to clinical details, who reviewed the raw motion-corrected images and corresponding stress perfusion map.

The diagnostic utility of ECG changes in detecting quantitative perfusion abnormalities was assessed. Diagnostic performance of ST-segment changes on ECG during adenosine testing at detecting minimum and mean sMBF thresholds (< 2.5, < 2.0, < 1.8, < 1.5, < 1.2 and < 1.0 ml/g/min) was evaluated at the myocardial territory level (anteroseptal, lateral, inferior). Because each participant contributed up to three territories, observations were clustered within patients. Sensitivity, specificity, positive predictive value (PPV), negative predictive value (NPV) and Youden’s J statistic (sensitivity + specificity − 1) were calculated for each threshold (23), with 95% CI estimated using cluster bootstrap resampling with 2000 resamples at the patient level to account for within-participant clustering using the boot package.

The receiver operating characteristic (ROC) curve was generated by plotting the sensitivity against (1-specificity) separately for mean and minimum sMBF across all thresholds, and area under the curve (AUC) was calculated as the area under the ROC curve.

A direct comparison with the in-built 3-lead trace was not undertaken, as this signal is acquired at monitoring bandwidth and is not intended for ST-segment or diagnostic analysis.

### Repeatability studies

To assess the measurement variability of in-bore resting ECG parameters, intra- and inter-observer variability studies were carried out on all 26 participants. In-bore QRS and QT duration measurements were repeated twice by D.F. 7 days apart (for intra-observer variability) and once separately by I.W. (for inter-observer variability), with both readers being blinded to the first readings and clinical data. Two-way mixed, single measures ICC (3, 1) was used to assess intra-observer reliability, and inter-observer reliability was assessed using the ICC based on a two-way random-effects model for single measurements (2, 1).

## Results

Baseline characteristics of the 26 participants are reported in Table 1 (34.6% female, 70.8 ± 13.0 years).

**Table 1:** Baseline characteristics of study participants

Clinical and demographic characteristics	Older age population-based volunteers (*n* = 20)	Younger healthy volunteers (*n* = 2)	Patients with known perfusion defects (*n* = 4)
Age (years)	76 ± 0	33.5 ± 0.7	63.5 ± 15.2
Female, *n* (%)	8 (40)	1 (50)	0
BSA (m^2^)	1.83 ± 0.19	1.81 ± 0.22	1.8 ± 0.2
Resting HR (beats per minute)	66 (61–77)	63.5 ± 7.8	64 (58–68)
Type 2 diabetes mellitus, *n* (%)	3 (15)	0	2 (50)
Hypertension, *n* (%)	9 (45)	0	1 (25)
High cholesterol, *n* (%)	4 (20)	0	1 (25)
Previous MI, *n* (%)	0 (0)	0	1 (25)
Ischaemic heart disease, *n* (%)	2 (10)	0	2 (50)
Alpha blocker *n* (%)	1 (5)	0	0 (0)
Angiotensin receptor blocker *n* (%)	2 (10)	0	2 (50)
Beta blocker *n* (%)	4 (20)	0	0 (0)
Calcium channel blocker *n* (%)	7 (35)	0	2 (50)
Chest pain on exertion *n* (%)	1 (5)	0	2 (50)
Dyspnoea on exertion *n* (%)	1 (5)	0	2 (50)
*CMR parameters*			
LVEDVI (ml/m^2^)	77.3 ± 14.7	83.5 ± 17.7	88.5 ± 16.7
LVEF (%)	65.6 ± 5.4	62 ± 4.2	57.3 ± 7.2
LVESVI (ml/m^2^)	26.4 ± 6.9	32.5 ± 10.6	38.0 ± 11.5
LVMI (g/m^2^)	50.8 ± 8.3	53.5 ± 4.9	65.5 (62.5–72.5)
MWT (mm)	10.0 ± 1.7	9.0 ± 0	12.5 ± 2.9
RVEDVI (ml/m^2^)	83.2 ± 17.3	72.5 ± 3.5	89.7 ± 12.5
RVEF (%)	59.8 ± 3.8	63 ± 2.8	49 (40.5–52.5)
LAAI (cm^2^/m^2^)	10.1 ± 2.7	11.5 ± 0.7	11.8 ± 3.3
Qualitative inducible perfusion defect *n* (%)	5 (25)	0	4 (100)
Global mean sMBF (ml/g/min)	2.0 (1.7–2.2)	3.7 ± 0.4	1.3 (1.1–1.6)
Global endocardial sMBF (ml/g/min)	1.9 (1.6–2.2)	3.9 ± 0.8	1.0 (0.8–1.3)
Global epicardial sMBF (ml/g/min)	1.9 (1.8–2.3)	3.6 ± 0.0	1.6 ± 0.8
*Out-of-bore reference 12-lead ECG parameters*			
RR interval (ms)	909 (777–983)	952 ± 117	941 (876–1038)
PR interval (ms)	189 ± 24	140 ± 22	152 ± 20
QRS duration (ms)	101 ± 19	82 ± 5	91 (89–101)
QT interval (ms)	361 ± 29	387 ± 29	406 ± 24
*In-bore resting 12-lead ECG parameters*			
RR interval (ms)	876 ± 109	1034 ± 64	967 ± 218
QRS duration (ms)	115 ± 14	91 ± 3	95 (91–110)
QT interval (ms)	371 ± 21	378 ± 37	385 (369–409)
*Adenosine-related side effects*			
Chest discomfort *n* (%)	10 (50)	2 (100)	3 (75)
Dyspnoea *n* (%)	11 (55)	2 (100)	2 (50)
Flushing *n* (%)	7 (35)	2 (100)	3 (75)
Transient AV block *n* (%)	0	0	0

Values are expressed as mean ± standard deviation, median (IQR) or counts (%)

BSA, body surface area; CMR, cardiovascular magnetic resonance; ECG, electrocardiogram; HR, heart rate; LAAI, left atrial area indexed to BSA; LVEDVI, left ventricular end diastolic volume indexed to BSA; LVEF, left ventricular ejection fraction; LVESVI, left ventricular end-systolic volume indexed to BSA; LVMI, left ventricular mass indexed to BSA; MI, myocardial infarction; MWT, maximum wall thickness; RVEDVI, right ventricular end-diastolic volume indexed to BSA; RVEF, right ventricular ejection fraction; sMBF, stress myocardial blood flow

### Feasibility and time to deploy

In-bore ECGs were successfully recorded in all cases. 12-lead ECGs were reconstructed for all resting ECGs, and for all but three adenosine ECGs. This was due to a technical (encryption) error at the time of recording meaning stored ECG files were corrupted and could not be retrieved for two participants, whilst lead detachment in one participant led to signal loss.

Additional pre-scan preparation time (beyond standard-of-care) to apply the electrodes was ~ 1 min per participant. Hardware connection occurred in parallel to the application of standard monitoring equipment, requiring no additional setup time. Post-processing for each participant took < 2 min. All hardware was reusable, except disposable single-use electrodes, and no equipment cleaning or disinfection was required between participants.

### Resting ECGs

#### In-bore vs. reference 12-lead ECGs

**Fig. 3:**
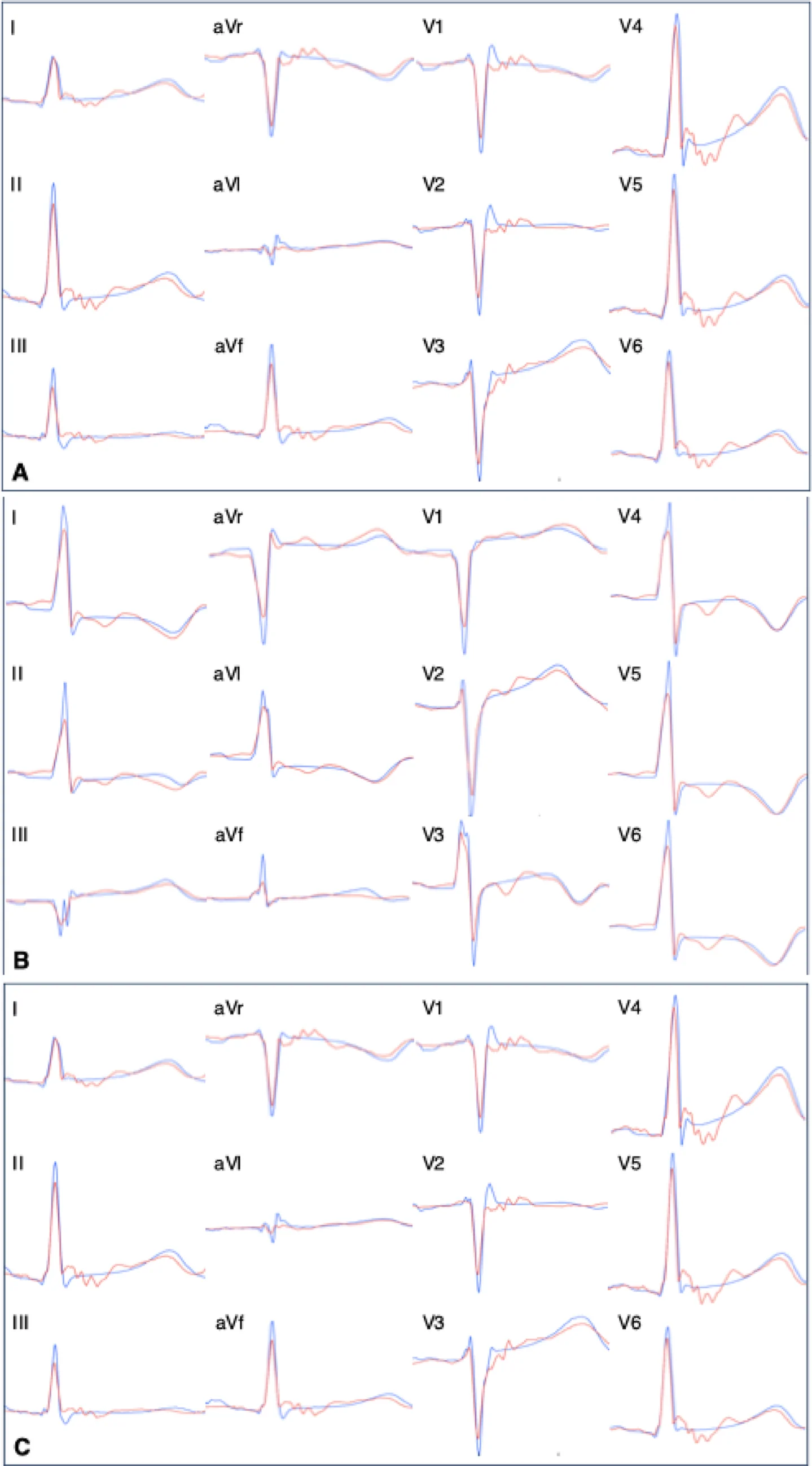
Example resting ECGs. Example reconstructed in-bore (red line) ECGs superimposed onto the reference (out-of-bore) resting ECGs (blue lines) for comparison, in 3 participants: **A**) healthy volunteer, 34-year-old male with no past medical history showing Pearson’s correlation coefficient (*r*) for all leads 0.93; **B**) 48-year-old male with known ischaemic heart disease, *r* for all leads 0.94; **C**) 76-year-old male with dilated cardiomyopathy, *r* for all leads 0.84. The in-bore ECGs appear smoother than the reference traces due to post-processing steps applied, including low-pass filtering

Comparing PQRST morphology, there was a median *r* of 0.81 (IQR 0.72–0.88) across all leads, and 0.89 (0.86–0.93) when selecting the lead with highest similarity for each participant. Representative examples of resting ECGs are presented in Fig. 3.

**Fig. 4:**
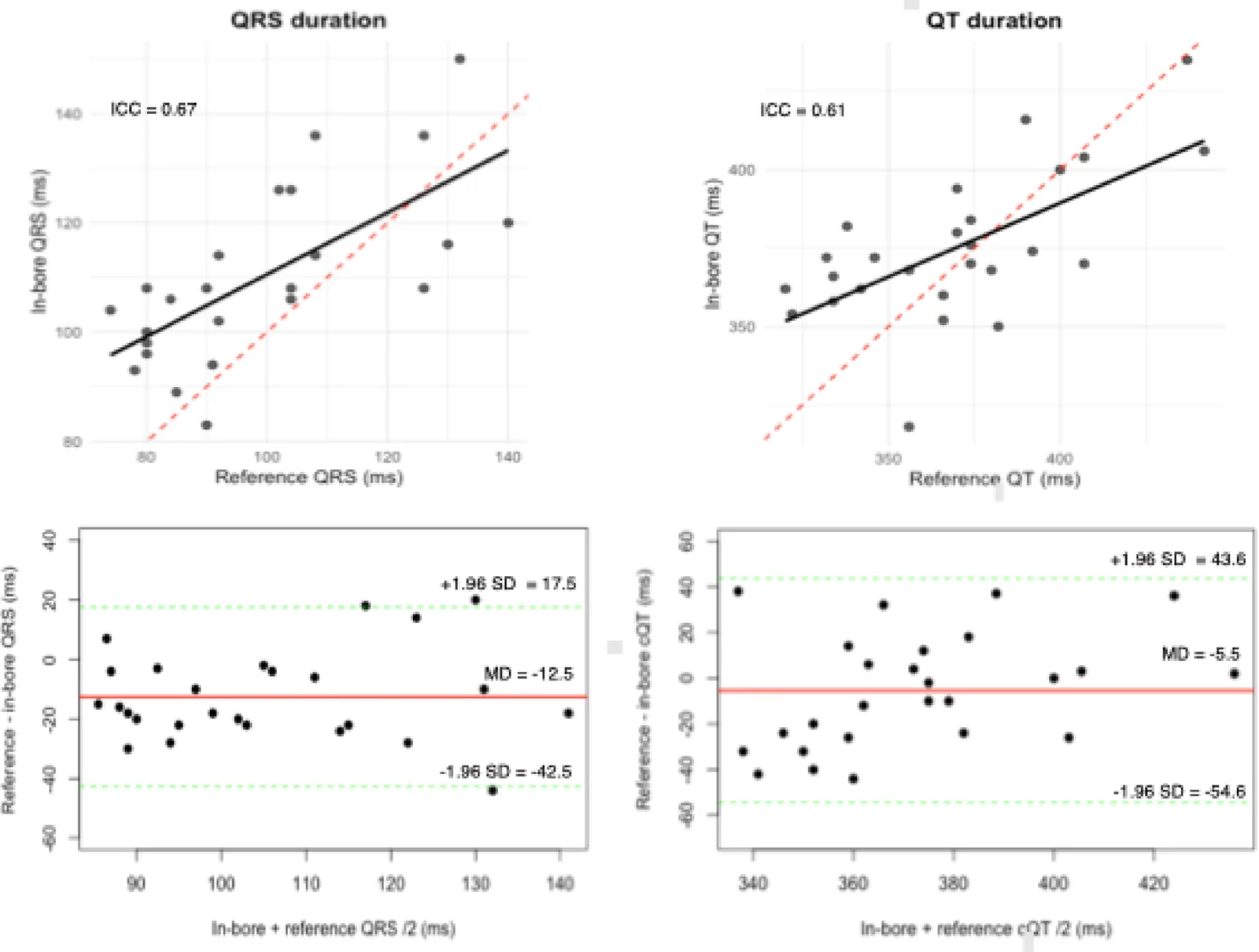
Intraclass correlation and Bland-Altman plots demonstrating reliability and agreement between reference and in-bore ECGs. ICC between reference and in-bore measurements was moderate (0.67) for QRS duration and QT duration (0.61). For ICC plots: solid black line = line of best fit, dashed red line = line of unity. *ICC*,* intraclass correlation coefficient; MD*,* mean difference; SD*,* standard deviation*

The MD in measured QRS duration was −12.5 ms (95% LOA − 42.5, 17.5 ms), with a moderate correlation (*r* = 0.69 [0.39–0.85], ICC = 0.67 [0.38–0.85] both *p* < 0.001). MD in QT interval was − 5.5 ms (95% LOA − 54.6, 43.6 ms) with moderate correlation (*r* = 0.64 [0.34–0.82], ICC = 0.61 [0.30–0.80] both *p* < 0.001). ICC and Bland-Altman plots are shown in Fig. 4.

Comparing *r* values between males and females, there was a MD of − 0.0656 (females − males) with a 95% CI of − 0.0996 to −0.0324, suggesting a statistically significant difference. There was a MD of 0.0137 comparing groups with a BMI above and below 30 kg/m^2^, with a 95% CI of −0.0350 to 0.0591, suggesting no significant difference.

#### Reproducibility studies

**Fig. 5:**
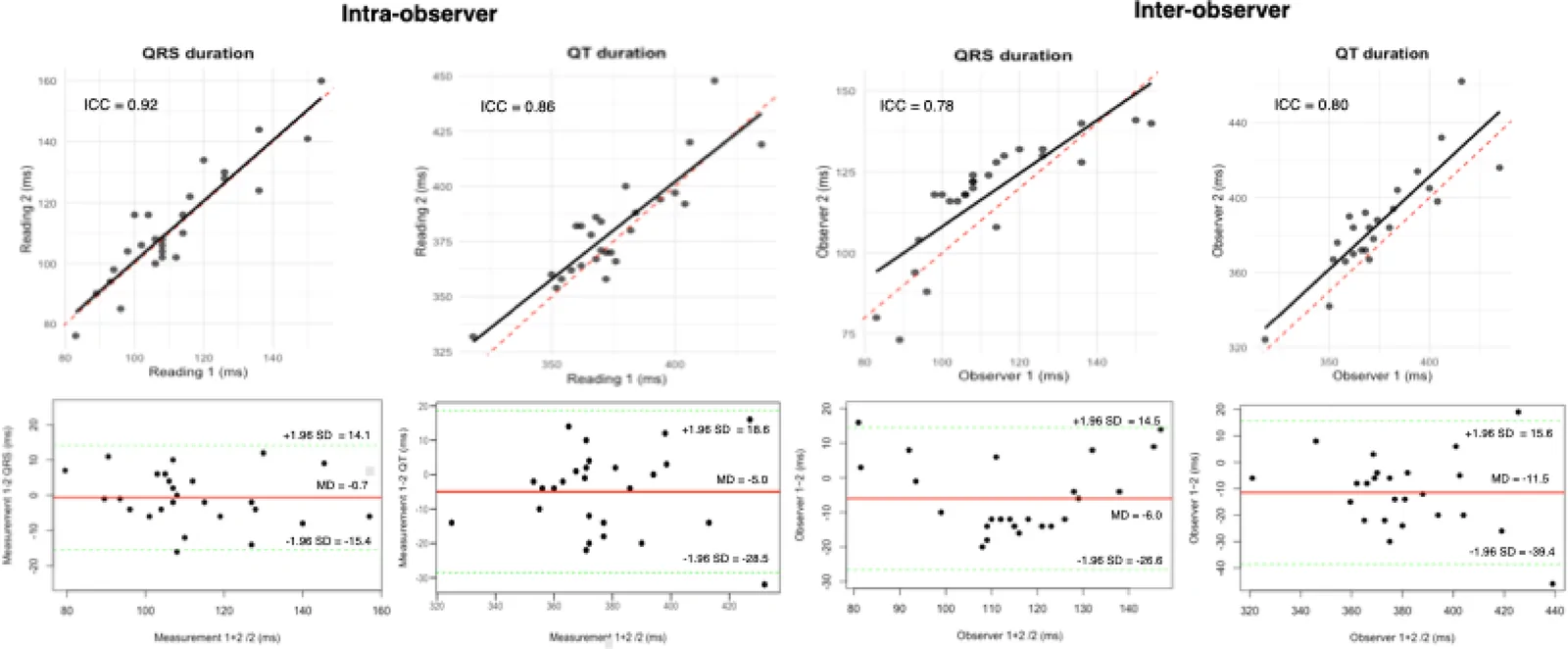
Intraclass correlation and Bland-Altman plots demonstrating intra- and inter-observer reliability and agreement of ECG parameters. ICC for intra-observer variability was strong for QRS (0.92) and QT (0.86) duration. ICC for inter-observer variability was strong for QRS (0.78) and QT (0.80) duration

ICC and Bland-Altman plots measuring the agreement of QRS and QT across intra- and inter-observer reproducibility studies are presented in Fig. 5. Correlation between QRS duration measurements was strong (intra-observer *r* = 0.92 [0.83–0.96], ICC = 0.92 [0.82–0.96]; inter-observer *r* = 0.82 [0.64–0.92], ICC = 0.78 [0.50–0.90], all *p* < 0.001). Correlation coefficients for QT duration were strong (intra-observer *r* = 0.87 [0.74–0.94], ICC = 0.86 [0.70–0.94]; inter-observer *r* = 0.87 [0.72–0.94], ICC= 0.80 [0.34–0.93], all *p* < 0.001).

MD was small when assessing intra-observer (QRS: −0.7 ms [95% LOA − 15.4, 14.1 ms]; QT: −5.0 ms [95% LOA − 28.5, 18.6 ms]) and inter-observer variability (QRS: −6.0 ms [95% LOA − 26.6, 14.5 ms]; QT: −11.5 ms [95% LOA − 39.4, 15.6 ms]).

### Adenosine ECGs

#### Healthy volunteer data: In-bore vs. reference ECGs during adenosine

**Fig. 6:**
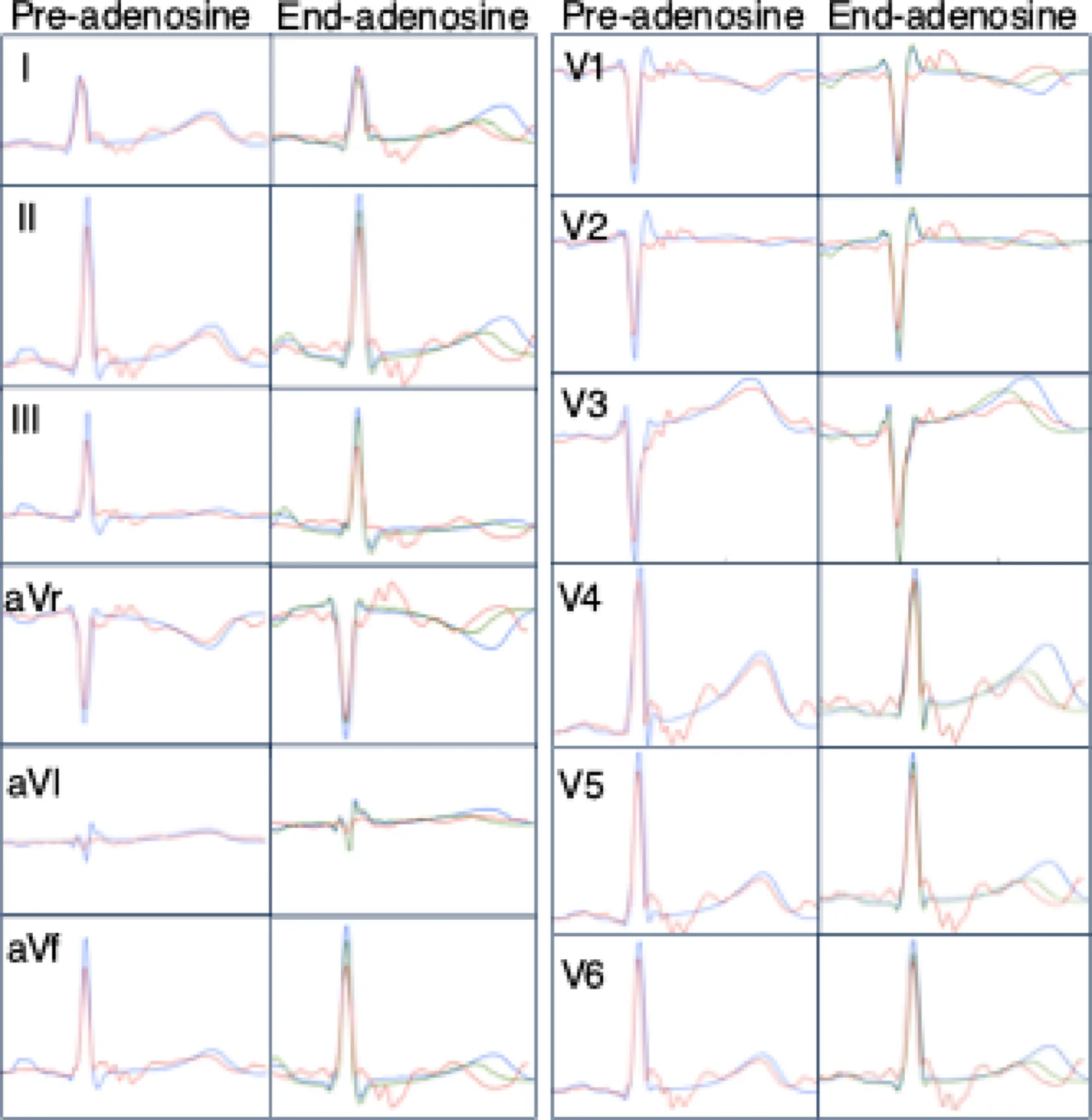
Comparison of in-bore and reference ECG signal at rest and at the end of adenosine infusion in a younger healthy volunteer. Left panel for each lead represents reference (blue line) and in-bore (red line) ECGs at rest (i.e. no adenosine). Right panel shows the resting reference out-of-bore ECG signal (blue line) with superimposed ECG signals collected at the end of the adenosine infusion both in-bore (red line) and out-of-bore (green line). As in resting recordings, reconstructed in-bore ECGs demonstrate a degree of smoothing relative to reference ECGs, reflecting post-processing applied for artefact suppression in the high-field MR environment

PQRST morphology showed good agreement prior to and during adenosine infusion: median (IQR) *r* across all leads was 0.93 (0.91–0.94) at rest, 0.88 (0.85–0.89) at the start, 0.83 (0.81–0.84) midway and 0.83 (0.82–0.85) at the end of the infusion (Fig. 6).

Measured QRS and QT durations at each time point are compared in Table 2.

**Table 2:** Exemplar healthy volunteer ECG metrics tracked throughout adenosine infusion

Parameter	Reference (ms)	In-bore (ms)
*QRS*		
Pre-adenosine	81	94
Early-adenosine	88	94
Mid-adenosine	84	82
End-adenosine	75	79
*QT*		
Pre-adenosine	397	408
Early-adenosine	369	375
Mid-adenosine	352	321
End-adenosine	335	322
*RR interval*		
Pre-adenosine	980	1157
Early-adenosine	670	986
Mid-adenosine	660	644
End-adenosine	660	629

Minor J point deviations were noted on the in-bore ECGs, but there were no significant detectable changes in ST segments or T waves. Mean global sMBF was 3.46 ml/min/g, and there were no segments with sMBF < 1.5 ml/g/min or visual perfusion defects.

#### In-bore adenosine ECGs in all participants

Reconstruction of adenosine ECGs was possible in 23 patients (saved files were corrupted for 2 patients and could not be retrieved, and for one patient lead detachment during recording led to loss of signal). Data from one further patient were excluded due to under-stressing, based on absence of hyperaemia and splenic switch-off. ST changes were detected in the anteroseptal leads in 3 participants (13.6%), in the inferior leads in 2 participants (9.1%), and in the lateral leads in 7 participants (31.8%). Concordance between symptoms at baseline and ST changes on reconstructed 12-lead ECGs are shown in the Supplementary appendix, Table S-1.

**Fig. 7:**
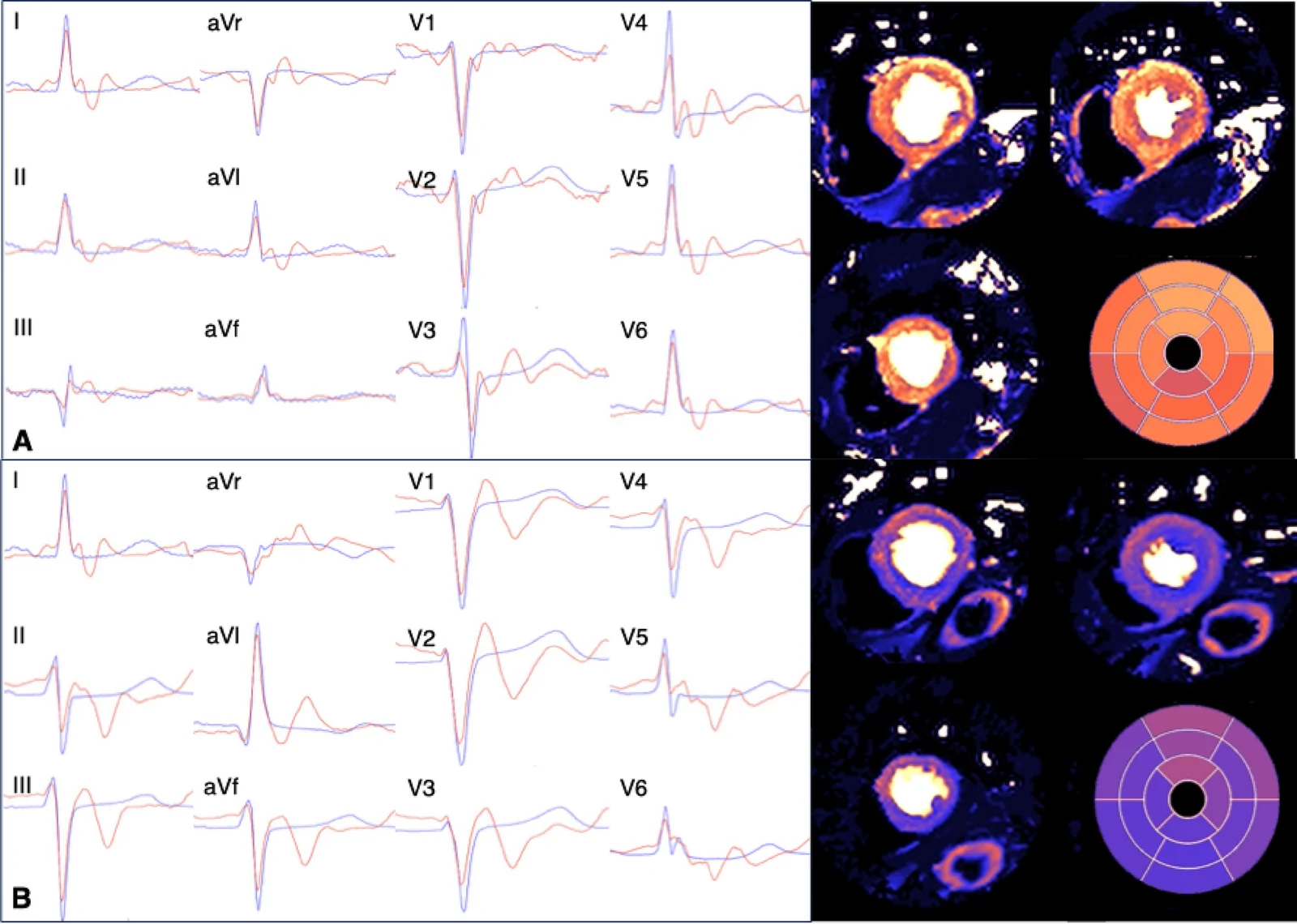
Exemplar ECGs with corresponding stress perfusion maps. Exemplar ECGs from 2 participants (blue line = resting out-of-bore reference ECG; red line = in-bore ECG during peak adenosine infusion). (**A**) 76-year-old patient with no past medical history. There are no inducible ischaemic changes on the in-bore ECG at peak adenosine or on quantitative stress perfusion maps. (**B**) 76-year-old patient with known ischaemic heart disease. In-bore ECG at peak adenosine shows dynamic T wave inversion in all ECG territories, with corresponding extensive inducible hypoperfusion, most marked in the inferior and septal territories

Exemplar in-bore ECGs with associated perfusion maps are shown in Fig. 7. On interpretation of qualitative perfusion maps, inducible perfusion defects were reported in 56 segments corresponding to 16 vascular territories in 8 participants. On quantitative stress perfusion mapping, sMBF was < 1.5 ml/g/min in 72 segments, or 22 vascular territories in 10 participants.

**Fig. 8:**
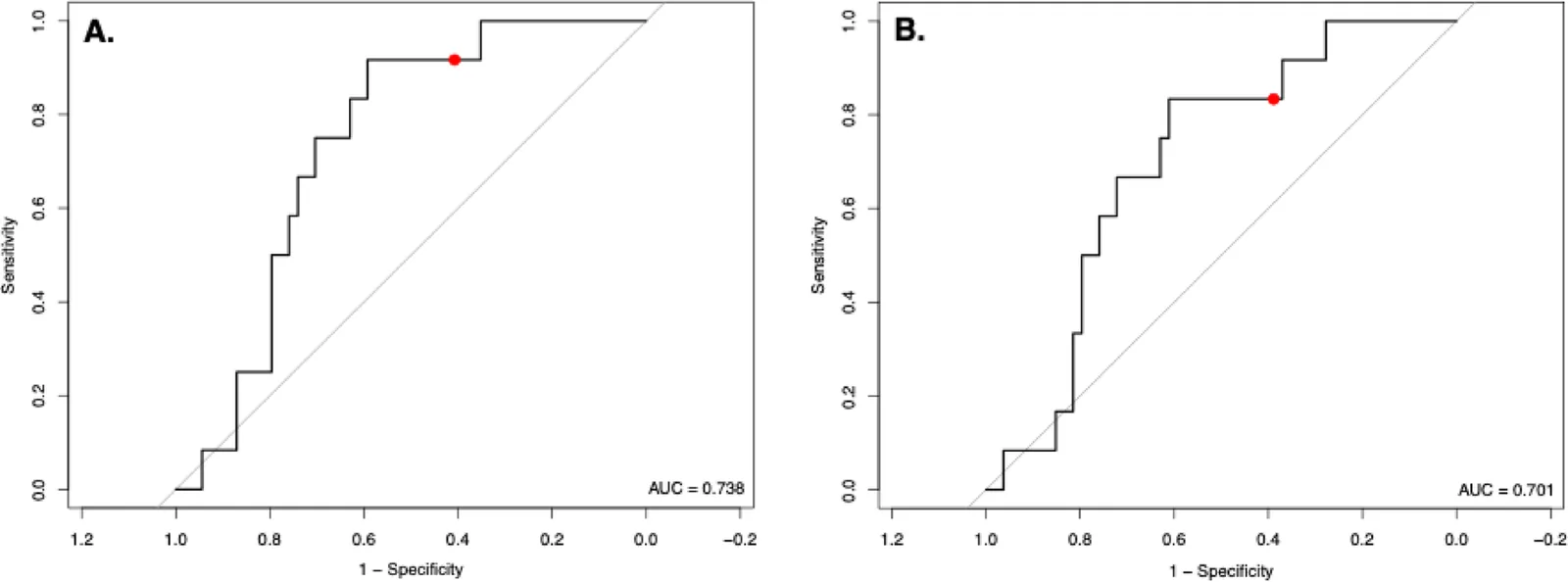
Receiver operating characteristics curves demonstrating model performance at identifying reduced sMBF per anatomical territory on the basis of minimum sMBF (**A**) and mean sMBF(**B**). Youden’s Index (red point) optimal threshold for ECG changes to detect reduced sMBF based on minimum sMBF is 1.75 ml/g/min (0.51 [0.20–0.75]), and 2.01 ml/g/min (0.43 [0.10–0.69]) for mean sMBF. *AUC*,* area under curve; sMBF*,* stress myocardial blood flow*

At all sMBF considered clinically reduced (< 1.5 ml/g/min), ischaemic changes on ECG had a high specificity and NPV for predicting reduced mean and minimum sMBF of the corresponding vascular territory (Table 3A, B). Detection of ECG abnormalities was superior at predicting minimum sMBF (AUC 0.74 [0.61–0.86]) compared with mean sMBF (0.70 [0.56–0.85]). Based on Youden’s Index, the optimal threshold for minimum sMBF was 1.75 ml/g/min (0.51 [0.20–0.75]), and for mean sMBF was 2.01 ml/g/min (0.43 [0.10–0.69]) (Fig. 8).

**Table 3:** Relationship between ECG changes during adenosine and (A) minimum or (B) mean stress myocardial blood flow per anatomical territory

Minimum sMBF threshold (ml/g/min)	Sensitivity % (95% CI)	Specificity % (95% CI)	PPV % (95% CI)	NPV % (95% CI)	Youden’s Index (95% CI)
< 2.5	100	18.5	21.4	100	0.19
	(100.0, 100.0)	(4.1, 35.4)	(7.9, 37.0)	(100.0, 100.0)	(0.04, 0.35)
< 2.2	100	29.6	24	100	0.29
	(100.0, 100.0)	(10.5, 50.9)	(9.3, 41.1)	(100.0, 100.0)	(0.11, 0.51)
< 2.0	91.7	35.2	23.9	95	0.27
	(66.7, 100.0)	(15.7, 55.9)	(7.7, 41.9)	(80.0, 100.0)	(-0.05, 0.53)
< 1.8	91.7	51.9	29.7	96.6	0.44
	(66.7, 100.0)	(30.9, 72.4)	(10.0, 51.4)	(87.0, 100.0)	(0.09, 0.70)
< 1.75	91.7	59.3	33.3	97	0.51
	(66.7, 100.0)	(39.6, 77.6)	(11.4, 57.7)	(90.0, 100.0)	(0.20, 0.75)
< 1.5	66.7	72.2	34.8	90.7	0.39
	(33.3, 90.0)	(55.4, 87.7)	(10.5, 62.5)	(80.0, 98.0)	(0.05, 0.69)
< 1.2	25	87	30	83.9	0.12
	(0.0, 60.0)	(72.5, 100.0)	(0.0, 100.0)	(67.9, 96.4)	(-0.14, 0.49)
< 1.0	16.7	87	22.2	82.5	0.04
	(0.0, 42.9)	(72.5, 100.0)	(0.0, 100.0)	(66.7, 94.6)	(-0.18, 0.32)
< 0.8	8.3	92.6	20	82	0.01
	(0.0, 33.3)	(83.6, 100.0)	(0.0, 50.0)	(67.2, 94.0)	(-0.13, 0.24)
PPV, positive predictive value; NPV, negative predictive value. Other abbreviations as in Table 1.					
Mean sMBF threshold (ml/g/min)	Sensitivity % (95% CI)	Specificity % (95% CI)	PPV % (95% CI)	NPV % (95% CI)	Youden’s Index (95% CI)
< 2.5	91.7	31.5	22.9	94.4	0.23
	(66.6, 100.0)	(12.8, 52.6)	(7.3, 40.5)	(77.8, 100.0)	(-0.08, 0.47)
< 2.2	83.3	44.4	25	92.3	0.27
	(50.0, 100.0)	(24.4, 65.0)	(8.1, 45.2)	(78.6, 100.0)	(-0.05, 0.56)
< 2.0	75	61.1	30	91.7	0.36
	(37.5, 100.0)	(43.2, 77.6)	(7.7, 53.1)	(80.6, 100.0)	(-0.04, 0.67)
< 1.8	66.7	70.4	33.3	90.5	0.37
	(20.0, 91.7)	(51.2, 88.1)	(5.9, 64.0)	(80.6, 97.9)	(-0.10, 0.69)
< 1.5	16.7	81.5	16.7	81.5	-0.02
	(0.0, 38.5)	(64.3, 94.9)	(0.0, 44.4)	(65.3, 94.1)	(-0.23, 0.22)
< 1.2	8.3	96.3	33.3	82.5	0.04
	(0.0, 33.3)	(91.2, 100.0)	(0.0, 50.0)	(68.2, 94.0)	(-0.05, 0.27)
< 1.0	0	96.3	0	81.2	-0.04
	(0.0, 0.0)	(91.2, 100.0)	(0.0, 0.0)	(67.2, 93.7)	(-0.09, 0.00)
< 0.8	0	98.1	0	81.5	-0.02
	(0.0,0.0)	(94.1, 100)	(0.0, 0.0)	(67.7, 93.8)	(-0.06, 0.00)

Abbreviations as per Tables 1 and 3A

Ischaemic ECG changes were associated with visible perfusion defects on qualitative assessment of stress perfusion cines and corresponding maps adjudicated in concordance by both readers, with 41.7% sensitivity (0.0**–**77.8%), 79.6% specificity (63.6**–**93.0%), 31.2% PPV (0.0**–**66.7%), 86.0% NPV (73.2**–**96.2%), Youden’s Index 0.21 (− 0.18, 0.59) and AUC 0.39 (0.24–0.55).

## Discussion

In this proof-of-principle study, we demonstrate the feasibility of integrating an external ECG device into a high-throughput 3 T clinical scanner. High-quality, in-bore 12-lead ECGs were safely recorded in all participants with minimal addition to workflow time. There was a strong correlation with reference ECGs when comparing waveform morphology.

Mean differences in QRS and QT durations were modest, although limits of agreement were wide, particularly for QT interval (-54.6, 43.6 ms), reflecting the effects of MHD artefact, filtering, and reconstruction. Although further improvements would be needed before this could be considered diagnostic-quality, the ability to measure QT intervals with reasonable reproducibility inside the MR bore may still be valuable for trend monitoring, safety surveillance, and research applications where relative rather than absolute changes are of interest.

Additionally, we undertook the first in-bore ECG recording during adenosine stress perfusion. In this cohort, where one third of participants had inducible perfusion defects, ischaemic ECG changes were associated with low sMBF and visible perfusion defects with high specificity and NPV, and relatively low sensitivity and PPV.

In our small cohort, we found a higher correlation between in-bore and reference ECG features in males than in females. We hypothesise this arises from greater variability in electrode position and artefact in females due to breast tissue. Lateral electrodes in females were placed under the breast to minimise interference, but it remains possible that placement of the coil may have resulted in changes in lead position. Owing to small participant numbers, multivariable analysis was not possible to ascertain if this effect could be explained by another variable. Further studies are needed to confirm these sex differences and to optimise recordings in females if proven.

The in-bore ECG recording process was compatible with the existing CMR scheduling, requiring no additional scan time, although it required additional steps beyond standard clinical practice. Our electrodes were placed alongside standard CMR cardiac monitoring (in this case, 4 electrodes), but could theoretically fully replace other cardiac monitoring to further streamline the process. Although we used 3 electrode patches, faithful reconstruction of 12-lead ECGs is possible with fewer leads (24) using linear transformation, further reducing preparation time.

Our current workflow requires an out-of-bore reference 12-lead ECG to permit in-bore ECG reconstruction, which may be impractical in a busy clinical environment. A potential solution may lie in the use of cycle-consistent generative adversarial networks (cycleGANs): such deep learning models can achieve automatic training of image-to-image translation without paired examples, and they have been successfully used to reconstruct ECGs with high fidelity (correlation > 90% (25)) after removing MHD effects, thus obviating the need for paired ground-truth ECGs.

Although our post-processing pipeline was fast (< 2 min per participant), in-bore ECGs cannot currently be implemented in real-time, which would be a pre-requisite for use during MR-guided interventions. Isaieva et al. proposed a hardware system which communicated with the Gadgetron for real-time reconstruction of images using physiological data from external devices including ECGs and respirometers. In one volunteer, the integrated ECG system was successfully used to gate a phase sensitive inversion recovery sequence at 3 T (26). Though in this instance the ECG was only used for R peak detection, clinical real-time application may be possible for 12-lead ECGs.

The safety and feasibility of collecting in-bore ECGs using a variety of systems has previously been reported in animal experiments and small healthy cohorts (Supplementary appendix, Table S-2). Dos Reis et al. used similar hardware to that presented herein (27), demonstrating heating of the system was below thresholds set by the International Electrotechnical Commission 60601-2-33 Standard (28). More recently, Kolandaivelu et al., compared accuracy of in-bore 12-lead ECGs recorded at 0.55 T, 1.5 T and 3 T (20). ECG electrodes with carbon-fibre leads were used with no additional shielding or post-processing. As expected, there was significantly lower median ECG error (defined as the difference in voltage between isoelectric lines) at 0.55 T compared to 1.5 and 3 T (0.14 mV vs. 0.41 mV vs. 0.81 mV respectively, *p* < 0.001), suggesting ECGs recorded inside a 0.55 T magnet may be interpreted without additional post-processing (29). Lower field strength reduces the MHD artefacts and the risk of radiofrequency heating, and recent data suggest that the quality of cardiac volumetric and functional assessment is comparable to that obtained at higher field strengths (30). However, few centres actually have a 0.55 T magnet in clinical use currently. Although interest has increased in lower field strengths in recent years, multiparametric mapping or stress perfusion imaging are less developed than they are at 1.5 or 3 T.

To our knowledge, ours is the first attempt at recording in-bore ECGs during adenosine infusion in humans. Kolandaivelu et al., measured ST segment changes during balloon-induced coronary occlusion in seven pigs, observing significant J point deviation at 1.5 and 3 T, limiting interpretation of ST segment changes. There was, however, good correlation between J points at 0.55 T after occlusion (*r* = 0.92), suggesting interpretation of ischaemia testing at lower field strengths may be possible (20). Dos Reis et al., performed Monte Carlo simulations of ischaemic and arrhythmic events, during which the mean correlation between in-bore and reference ECG signals remained high at > 85% (25).

### Potential applications

If the challenges imposed by the MR environment can be overcome, in-bore ECGs have several potential applications. Applied in real-time, a cleaner signal could enhance patient monitoring and in-bore safety. Improved R-peak detection could result in better ECG triggering, with improved image acquisition and reduced scan time. 12-lead ECGs, allowing localisation of arrhythmia to clinical territories, could support CMR-guided interventions such as ablation. Additionally, in-bore ECG recordings for electrocardiographic imaging (31) (ECGI) would enhance the accuracy of reconstruction maps.

Our findings of high specificity and NPV for detecting ischaemia correlate with previous data on exercise treadmill ECG from the SCOT-HEART trial, which had a low sensitivity (39%) and PPV (38%) but high specificity (91%) for detecting obstructive coronary artery disease on invasive angiogram (32). Whilst in-bore ECGs are therefore not a useful ‘rule in’ test for ischaemia, their addition may be useful to aid interpretation of perfusion mapping e.g. in guiding clinicians if there is uncertainty about whether a lesion represents a genuine defect vs. artefact, or supporting visual (qualitative) rulings in the event of mapping reconstruction errors, which still occur in > 1% of stress CMR scans (33).

Neurocardiology and heart-brain imaging rely on ECG signals to align brain activity to the cardiac cycle. Changes in QT duration (34) and T wave morphology (35) can occur with emotional stress but cannot currently be monitored during brain imaging. Concomitant monitoring of QT/ T wave changes with functional MRI of the brain may offer new insights into heart-brain interactions across cardiovascular, neurological and psychiatric conditions.

### Study limitations

Our study was designed to address the hardest challenge of recording in-bore 12-lead ECG recordings at 3 T, meaning that results obtained are not immediately generalizable to other field strengths, or vendors. However, smaller MHD effects at 1.5 and 0.55 T mean we anticipate better results than those presented herein.

This study is limited by its small sample size, leading to wide confidence intervals around the diagnostic performance estimates (Table 3). Although this is the largest reported human cohort with in-bore ECGs to-date, and the first group with recordings during adenosine, there were relatively few patients with confirmed inducible perfusion defects (*n* = 8), precluding sub-group analysis. Future work is needed to explore the clinical utility of in-bore stress perfusion ECG in discriminating between epicardial and microvascular disease, or between 3-vessel disease and global hypoperfusion. Another limitation is the small number of abnormal baseline ECGs – only two patients were in atrial fibrillation, five had bundle branch block and one had T wave inversion. As a proof-of-concept study, our findings should be interpreted as hypothesis-generating, and larger prospective cohorts are needed to confirm the diagnostic performance characteristics reported here.

ECG reconstruction relied on a subject-specific linear transformation derived from the (resting) reference ECG which was applied to in-bore resting and adenosine stress ECGs. While electrode position remains fixed, ischaemia and dynamic physiological changes may alter cardiac electrical vectors and repolarisation patterns, raising concerns regarding the validity of a static reconstruction matrix under stress conditions. In this proof-of-concept study, correlation between in-bore and out-of-bore waveform morphology in healthy volunteers remained high across all stress time points, with no clear spurious ST/– T wave changes (except minor J point deviations). However, adaptive reconstruction strategies which incorporate physiological changes may further improve performance in future studies.

When appraising inducible perfusion defects and corresponding ECG abnormalities, we made standard clinical assumptions relating to underlying anatomy and arterial supply. Of course, patient-specific variations mean that co-localisation of ECG and perfusion mapping territories may vary in some cases (36). However, without prior coronary imaging, we were unable to fully evaluate this and applied a pragmatic approach using territories typically used in clinical practice, which will apply to most participants.

Lastly, as this was a feasibility study focused on establishing the viability of 12-lead ECG reconstruction, evaluation of its application to real-time ECG-triggered gating was outside the scope of this work and represents an important direction for future research.

## Conclusion

This proof-of-principle study shows that integration of an external ECG device into a high-throughput 3 T clinical MR-scanner is feasible, and capable of recording 12-lead ECGs with good morphological correlation to reference ECGs. In-bore adenosine ECGs have a high specificity and NPV, but low sensitivity and PPV for predicting inducible ischaemia. Future work is needed to understand the potential clinical utility of in-bore 12-lead ECGs for improved patient monitoring, ECG-gating and the interpretation of stress perfusion mapping.

## Supplementary Information

Below is the link to the electronic supplementary material.

Supplementary Material 1

## Data Availability

NSHD and MyoFit46 data is available from https://nshd.mrc.ac.uk/. For MyoFit46 collaborations, please access http://www.myofit46.com/. The datasets generated and/or analysed during the current study are available from the corresponding author on reasonable request.

## Declarations

### Permissions

Figures were created by the authors (DF).
