# Supplementary material for: Feasibility of 12-lead ECG reconstruction at rest and during adenosine stress perfusion inside a 3-Tesla MR scanner
Source: Int J Cardiovasc Imaging. 2026 Jul 21;42(10):1983–98. doi: 10.1007/s10554-026-03772-z (PMC13646000; doi:10.1007/s10554-026-03772-z)
Supplement: Supplementary file 1 [file 10554_2026_3772_MOESM1_ESM.docx]

**Supplementary material for:**

**Feasibility of 12-lead ECG reconstruction at rest and during adenosine stress perfusion inside a 3-Tesla MR scanner**

Debbie Falconer^1^, Maroua Mehri^2^, Mohammed Bhiri^2^, Matt Webber^1,3^, Isabelle Whitmore^1,4^, Emma Martin^1^, Lorena Topala^1^, Giovanni Vagliani^2^, Franca Morselli^1^, Haytham Shah^4^, Swapnanil De^4^, Thomas A Treibel^1,3,5^, Michele Orini^6^, Nish Chaturvedi^1^, Iain Pierce^1,3^, Peter Kellman^1,8^, Pier D Lambiase^1,3^, James C Moon^1,3,5^, Alun D Hughes^1^, Freddy Odille^7^, Guillaume Calmon^2^, Gabriella Captur^1,9^

**Author affiliations**

1. UCL Institute of Cardiovascular Science, University College London, London, UK

2. Epsidy, Rue du Morvan, Vandoeuvre-les-Nancy, France.

3. Barts Heart Centre, West Smithfield, London, UK

4. UCL Medical School, University College London, London, UK

5. Chenies Mews Imaging Centre, The Roger Williams Building, London, UK

6. King’s College London, Strand, London, UK

7. IADI Laboratory, Institut National de la Santé et de la Recherche Médicale. Rue du Morvan, Vandoeuvre-les-Nancy, France.

8. National Heart, Lung, and Blood Institute, National Institutes of Health, Bethesda, Maryland, USA

9. The Inherited Cardiac Conditions Unit, Royal Free London NHS Foundation Trust, London, UK

**Further methods**

*Signal processing and ECG reconstruction*

Reconstruction of in-bore 12-lead ECGs required calibration using the reference out-of-bore resting ECG signal (herein termed $V_{12}$). The in-bore ECG signals (termed $V_{Network}$) were considered linear combinations of the reference 12-lead ECG used in the calibration.

First, the signal quality of the two ECGs was enhanced by applying denoising pipelines.

For $V_{12}$, the following pipeline was applied:

1) Signal resampling to 500 Hz (from 600 Hz);
2) Third-order bandpass filter (frequency range of 0.05-150 Hz);
3) Notch filter and a high-pass filter (0.05 Hz) to remove baseline wandering.

For $V_{Network}$, the following filtering pipeline was applied:

1) Median filter at 16 kHz;
2) Signal resampling to 500 Hz;
3) High-pass filter at 2 Hz;
4) Kalman filter, a first-order recursive filter that operated in two steps. The first step was the prediction phase, where the next sample was estimated using a linear model based on the previous sample and an adaptive estimate of the signal’s covariance. In the second step, the update phase, the measured sample was compared to the predicted value, taking into account the noise—assumed to be Gaussian—and added to the true value;
5) Low-pass filter at 20 Hz.

Unlike hardware-limited monitoring systems, the *V_12_* signals were acquired using broadband hardware with a high sampling frequency (16 kHz). This high-fidelity acquisition ensures that the underlying morphology, including the ST-segment, was captured without the hardware-level attenuation or 'smearing' typical of lower-quality devices. While a 20 Hz low-pass filter was applied during post-processing to suppress high-frequency artifacts and facilitate automated analysis, the raw high-quality data remains preserved. Furthermore, the 0.05 Hz high-pass filter was strictly maintained according to diagnostic standards to ensure baseline stability, confirming that any observed ST-segment deviations were physiological and not the result of baseline wander.

Subsequently, cardiac cycle templates for two ECG acquisitions were constructed for $V_{Network}$ and $V_{12}$ by applying the following steps:

1) An R-peak detection algorithm (1) was applied to the entire signal;
2) The signal was sliced into 10 cardiac cycles;
3) A median cardiac cycle was estimated from the 10 cardiac cycles.

Median cardiac cycles for the two ECG acquisitions constituted the templates $V_{12}$ and $V_{Network}$ used in the reconstruction algorithm.

Next, $M$ matrix was constructed, representing the linear combination between the median cardiac cycles of the leads of the two ECG acquisitions using the Tikhonov regularization (2). Finally, a linear transformation was applied to the whole in-bore ECG signal, by multiplying the signals of the non-standard leads of the acquired in-bore ECG signal by the $M$ matrix. Linear transformation has been shown to produce high quality synthesised ECGs from smaller lead numbers (3). This multiplication was performed sample by sample and carried out in quasi real-time, represented by the following equation:

$$V_{12}\left( t_{n} \right)=V_{Network}\left( t_{n} \right)*M$$

**Table S-1:** Concordance between presenting symptoms and ST-segment changes on reconstructed 12-Lead ECG.

| **Presenting symptom** | **ST changes present (number of participants)** | **ST changes absent (number of participants)** |
| --- | --- | --- |
| Chest pain on exertion (*n* = 2) | 1 | 1 |
| Shortness of breath on exertion (*n* = 4) | 1 | 3 |
| Asymptomatic (*n* = 17) | 5 | 12 |

22 participants with reconstructed 12-lead ECGs included. One participant experienced both chest pain and shortness of breath at baseline.

**Table S-2**. Summary of studies to-date recording or reconstructing in-bore 12-lead ECGs.

| **Author, year** | **Field strength (T)** | **Cohort** | **System design features** | **Results** |
| --- | --- | --- | --- | --- |
| Krug, 2011 (4) | 3 | 1 HV | - 10 electrodes in conventional placement - Independent component analysis applied to estimate underlying signal | - Significant errors seen between filtered and clean ECGs |
| Tse, 2014 (5) | 1.5 | 10 HV  4 arrhythmia patients | - 10 electrodes in conventional placement - Coaxial shield over low impedance leads with DC blocking capacitors - Gradient ‘RF Blanking’ hardware to filter noise - Real-time processing and MHD filter | - Cross correlation index with reference ECG:   Healthy subjects 96.2%; patients 94.5%   - Root mean square error:   Healthy subjects 2.6%; patients 2.3% |
| Gregory, 2015 (6) | 3 | 2 HV | - 10 electrodes in conventional placement - Single lead placed across left common carotid artery to measure MHD - Carotid MHD trained filter to create ‘clean’ ECG | - Mean cross correlation 0.81 all leads between in-bore and reference ECG |
| Dos Reis, 2019 (7) | 1.5, 3 | 4 HV | - 4 electrodes each consisting of 3 measurement and 1 reference electrode with short leads connected to a signal module for signal amplification and digitalisation. - Subject-specific matrix is applied to reconstruct 12-lead ECG (so requires out-of-bore ECG) | Waveform similarity:   - 1.5T:   PQRST wave: 80.1%; QRS wave 93.7%   - 3T:   PQRST wave 64.6%; QRS wave 91.1% |
| Kolandaivelu, 2024 (8) | 0.55, 1.5, 3 | 5 HV at rest and with ergometer exercise  7 pigs with induced ischaemia | - 10 electrodes in conventional placement, carbon fibre electrodes with no additional shielding, and no post-processing | ECG error, median (range)   - 0.55T 0.14mV (0.10-0.15) - 1.5T 0.41mV (0.36-0.49) - 3T 0.81mV (0.57-0.88) |

*ECG, electrocardiogram; HV, healthy volunteer; MHD, magnetohydrodynamic effect, RF, radiofrequency, T, Tesla*

**Supplementary Appendix References**

1.	Mehri M, Calmon G, Odille F, Oster J. A Deep Learning Architecture Using 3D Vectorcardiogram to Detect R-Peaks in ECG with Enhanced Precision. Sensors (Basel). 2023;23(4).

2.	Cluitmans MJM, Clerx M, Vandersickel N, Peeters RLM, Volders PGA, Westra RL. Physiology-based regularization of the electrocardiographic inverse problem. Med Biol Eng Comput. 2017;55(8):1353-65.

3.	Tomasic I, Trobec R. Electrocardiographic systems with reduced numbers of leads-synthesis of the 12-lead ECG. IEEE Rev Biomed Eng. 2014;7:126-42.

4.	Krug JW, Rose G. Magnetohydrodynamic distortions of the ECG in different MR scanner configurations.Computing in cardiology;2011;Hangzhou, China.

5.	Tse ZT, Dumoulin CL, Clifford GD, et al. A 1.5T MRI-conditional 12-lead electrocardiogram for MRI and intra-MR intervention.Magn Reson Med.2014;71(3):1336-47.

6.	Gregory TS, Oshinski J, Schmidt EJ, Kwong RY, Stevenson WG, Ho Tse ZT. Continuous Rapid Quantification of Stroke Volume Using Magnetohydrodynamic Voltages in 3T Magnetic Resonance Imaging. Circ Cardiovasc Imaging. 2015;8(12):e003282.

7.	Dos Reis JE, Soullie P, Oster J, et al. Reconstruction of the 12-lead ECG using a novel MR-compatible ECG sensor network.Magn Reson Med.2019;82(5):1929-45.

8.	Kolandaivelu A, Bruce CG, Seemann F, et al. Evaluation of 12-lead electrocardiogram at 0.55T for improved cardiac monitoring in magnetic resonance imaging.J Cardiovasc Magn Reson.2024;26(1):101009.
